# Driving style diversity in highway weaving areas: A drone-based analysis of population distribution patterns and operational parameter relationships

**DOI:** 10.1371/journal.pone.0336040

**Published:** 2025-12-03

**Authors:** Zhibing Yuan, Zhenghua Huang, Xin Su, Peng Fu

**Affiliations:** 1 School of Foreign Studies, Yiwu Industrial & Commercial College, Jinhua, China; 2 Department of Traffic Operations, Shanxi Railway Vocational and Technical College, Taiyuan, China; 3 School of Industrial Affiliate, Xiangyang Auto Vocational Technical College, Xiangyang, China; Nanjing Forestry University, CHINA

## Abstract

Driving style heterogeneity significantly influences traffic safety and efficiency in highway weaving areas, yet how operational parameters systematically shape population-level behavioral patterns remains unclear. This study examines the relationships between weaving area operational characteristics and driving style population distribution patterns using drone-collected trajectory data from seven highway weaving areas, obtaining 12,707 complete vehicle trajectories. Six driving style features incorporating speed and acceleration metrics were extracted, and Gaussian Mixture Model clustering identified three distinct driving style types: aggressive (23.5%), moderate (58.9%), and conservative (17.6%). Population distribution characteristics were quantified using Shannon entropy, Gini coefficient, and dominance index, revealing significant variations across weaving areas with Shannon entropy values ranging from 0.376 to 0.703. Statistical analysis demonstrated that weaving complexity exhibited strong positive correlation with Shannon entropy (r = 0.909, p = 0.005), while headway time showed significant negative correlation (r = −0.760, p = 0.047). A critical threshold of 300m was identified for weaving length effects on population distributions. Machine learning-based driving style classification models incorporating 17-dimensional features achieved 95.4% identification accuracy, with lateral acceleration contributing the highest feature importance (35.5%) and operational parameters accounting for 31.4% of total feature significance. The findings reveal that operational parameters correlate with driving style distributions through hierarchical patterns where geometric parameters are associated with spatial constraints, traffic flow parameters provide dynamic regulation, and weaving task parameters create behavioral differentiation. This research contributes to understanding how infrastructure and traffic conditions systematically shape collective driver behavior patterns, offering new approaches for traffic management optimization in intelligent transportation environments.

## Introduction

Highway weaving areas, as critical sections where vehicles merge into and diverge from traffic streams, represent important bottlenecks and safety hazards in transportation systems. In these areas, drivers must complete complex lane-changing maneuvers within limited spatiotemporal constraints, creating significant traffic conflicts and operational bottlenecks [1]. Research consistently demonstrates that weaving areas experience substantially higher accident rates and reduced capacity compared to basic highway segments, with studies showing that the coexistence of merging, diverging, and through traffic creates significantly elevated collision risks [2]. Particularly noteworthy is that driving style diversity is especially pronounced in weaving areas, where different drivers exhibit distinctly different behavioral patterns when facing the same operational environment. This behavioral heterogeneity has significant impacts on the overall operational characteristics of weaving areas [3,4]. Therefore, in-depth research on the correlations between weaving area operational parameters and driving style population distribution is of great theoretical and practical significance for understanding traffic behavioral patterns and optimizing weaving area operational management.

In recent years, driving style identification research has evolved from early questionnaire-based approaches [5] to automatic identification based on actual driving data [6], with machine learning technology applications continuously improving identification accuracy [7,8]. Existing research primarily focuses on individual driving style identification and classification, categorizing driving styles into conservative, moderate, and aggressive types [9], and analyzing behavioral characteristic differences among drivers of different styles [10]. These studies have found that aggressive drivers tend to choose smaller gaps for lane changes, conservative drivers more cautiously wait for larger safety gaps, and moderate drivers fall between the two extremes while flexibly adjusting strategies according to situations [11].

In terms of weaving area operational characteristics research, scholars have gradually expanded from geometric parameters and traffic flow characteristics [12] to include analysis of driving behavioral factors [13], progressively recognizing that driver behavioral heterogeneity is an important dimension for understanding weaving area operational patterns. Existing research has found that weaving area operational parameters (such as weaving length, traffic density, vehicle composition, etc.) significantly influence drivers’ behavioral choices and traffic flow characteristics [14]. The development of drone technology has provided new data acquisition methods for traffic behavior research, capable of providing large-scale, high-precision trajectory data [15], offering technical support for simultaneously observing multiple drivers’ behavioral interactions and population distribution characteristics [16].

However, existing research has obvious limitations, primarily manifested in three aspects. First, traditional driving style research mainly focuses on individual driver style identification and individual behavioral characteristic analysis, concentrating on how to accurately identify and predict individual driving behavior [17], but rarely studies the spatial distribution patterns and clustering characteristics of different style drivers from a population perspective. Second, although weaving area operational analysis research recognizes the importance of driving behavior, it mostly analyzes operational efficiency and capacity from a macroscopic traffic flow perspective [18], lacking understanding of the microscopic patterns of how operational parameters shape driving style population distribution. Third, existing research lacks a systematic theoretical framework that organically integrates operational parameters, driving style population distribution, and key characteristics, neglecting questions regarding how weaving area operational parameters systematically influence driving style population distribution patterns and how key characteristics of driving style identification are modulated by the operational environment.

In view of this, this study aims to systematically analyze the correlations between weaving area operational parameters and driving style population distribution from the perspective of population distribution patterns. The main contributions of this research are threefold. First, it establishes a population distribution analysis framework using Shannon entropy, Gini coefficient, and dominance index to quantify collective behavioral patterns, shifting the research focus from individual driving style identification to population-level characteristics. Second, by collecting high-precision drone trajectory data from seven typical weaving areas with different operational characteristics and employing Gaussian Mixture Model clustering, this study systematically reveals multi-level relationships between operational parameters and driving style distributions, including the effects of geometric parameters, traffic flow parameters, and weaving task parameters. Third, through constructing machine learning classification models, this research identifies key characteristics affecting driving style classification and demonstrates the significant role of operational parameters in shaping driving behaviors, validating the context-dependent nature of driving styles. These findings provide theoretical support for scientific operational management and intelligent control of weaving areas.

The remainder of this paper is organized as follows. Section 2 describes the methodology including study area selection, data collection procedures, driving style feature system construction, clustering analysis methods, population distribution quantification approaches, and machine learning modeling framework. Section 3 presents the results on driving style identification, population distribution characteristics across weaving areas, relationships between operational parameters and distribution patterns, and feature importance analysis. Section 4 discusses the practical implications, broader applications, and study limitations. Section 5 concludes with key findings and recommendations for future research.

## Methods

### Study areas and data collection

This study selected 7 typical highway weaving areas as research subjects from Yunnan, Guangdong, Jiangsu provinces and Shanghai municipality in China, representing diverse geographic conditions including mountainous and plain terrain. The selected sites cover different types of weaving areas with various geometric configurations and traffic characteristics. Drone aerial photography technology was employed to collect video data during daytime hours (9:00–17:00) under clear weather conditions with good visibility on weekdays, with flight altitude maintained at 500m to ensure complete coverage of the weaving areas. While environmental factors such as weather, visibility, and time of day can influence driving behavior, the controlled collection conditions minimize confounding effects and allow the analysis to focus on operational parameter relationships.

No specific permits were required for this observational study. The research involved non-intrusive observation of public traffic behavior on public highways using drone technology in compliance with Civil Aviation Administration of China (CAAC) regulations for unmanned aerial vehicle operations. All selected study sites were located outside CAAC-designated no-fly zones, and all drone operations were conducted under CAAC’s regulatory framework for civil unmanned aerial vehicles, maintaining appropriate altitude (500m) and safety distances from active traffic. No intervention or manipulation of traffic conditions was performed, and no personally identifiable information was collected.

DataFromSky software was used for video processing and trajectory extraction, which automatically identifies vehicle targets and reconstructs motion trajectories based on computer vision technology. The system extracts trajectory coordinates at a temporal frequency of 3 Hz (0.33-second intervals), capturing vehicle positions, speeds, and accelerations frame-by-frame. Geographic information registration was performed using ground control points to convert pixel coordinates to real-world coordinates with spatial measurement accuracy of approximately 0.06 m (1.12% relative error) based on validation against known road markings. Vehicle type identification accuracy was validated at 96.3% through comparison with manual annotation.

Trajectory data preprocessing involved three systematic steps to ensure data quality. First, completeness filtering removed trajectories that did not traverse the entire weaving section from entry to exit. Second, outlier detection and smoothing were applied: anomalous speed and acceleration values were identified using the 3σ criterion and corrected through local averaging or linear interpolation, followed by moving average smoothing with a 5-frame window to reduce random noise. Third, spatial alignment was performed by calculating cumulative distances referenced to characteristic points (e.g., diverging or merging locations) and resampling parameters at 1-meter intervals to enable cross-path comparisons at identical spatial positions. After these preprocessing steps, a total of 12,707 complete vehicle trajectories were obtained. The basic information of each weaving area is shown in Table 1, including lane configuration, geometric parameters, speed limits, main operating parameters such as Heavy Vehicle Ratio (HVR), Diverging Ratio (DR), and Merging Ratio (MR), and the number of trajectories collected at each site. Aerial photographs of each weaving area are also provided in Table 1 to illustrate the geometric configurations and traffic conditions at each study site.

**Table 1:** Basic Information of Data Collection Segments.

Zone	Ramp-Mainline-Ramp Lane Number	Weaving Length (m)	Mainline Design Speed (km/h)	Number of trajectories	Average Headway	Zone Average HVR	Zone Average DR	Zone Average MR
1	1-3-2	280	120	2068	1.270	0.199	0.073	0.223
2	2-3-2	240	100	2798	1.177	0.212	0.199	0.272
3	2-3-2	200	100	1639	1.313	0.177	0.212	0.226
4	2-3-1	630	80	142	1.490	0.024	0.068	0.394
5	2-3-2	500	80	1204	1.403	0.029	0.393	0.057
6	1-3-2	240	80	2623	2.017	0.082	0.123	0.093
7	2-3-1	220	80	2233	2.089	0.051	0.108	0.144

To systematically analyze the relationships between operational parameters and driving style distributions, weaving area operational parameters were categorized into three hierarchical levels based on their functional roles and temporal characteristics. Geometric operating parameters include physical design elements that remain fixed over time, specifically weaving length and speed limit, which establish the fundamental spatial and regulatory constraints of the facility. Traffic operating parameters encompass dynamic flow characteristics that vary with traffic conditions, including average headway time and heavy vehicle ratio (HVR), which reflect the instantaneous traffic composition and density. Weaving task parameters describe the operational complexity arising from vehicle path choices, specifically diverging ratio (DR), merging ratio (MR), and weaving complexity (defined as DR + MR), which quantify the proportions of vehicles performing specific weaving maneuvers within the section. This three-level categorization enables systematic examination of how infrastructure design, traffic flow dynamics, and operational task demands independently and collectively influence driving style population distributions.

### Driving style feature system construction

A multidimensional driving style feature system was constructed, containing 6 trajectory behavioral features: average speed $\bar{v}$ , speed standard deviation $\sigma_{v}$, average longitudinal acceleration $a_{tan}$, maximum longitudinal acceleration $a_{tan\_max}$, average lateral acceleration $a_{lat}$, and maximum lateral acceleration $a_{lat\_max}$. The feature calculation formulas are:


$$\bar{v}=\frac{\text{1}}{n}\sum_{i=1}^{n}v_{i},\;\sigma_{v}=\sqrt{\frac{\text{1}}{n-1}\sum_{i=1}^{n}{\left(v_{i}-\bar{v}\right)}^{2}}$$ (1)

$$a_{tan}=\frac{dv}{dt},\;a_{lat}=\frac{v^{2}}{R}$$ (2)

where $v_{i}$ represents instantaneous speed, n represents the number of sampling points, and R represents the radius of curvature. These features characterize drivers’ behavioral patterns from two dimensions of speed control and acceleration operation, providing a quantitative foundation for subsequent driving style identification.

### Driving style identification and classification

Gaussian Mixture Model (GMM) was employed for clustering analysis of driving style features. The 6 trajectory behavioral features (average speed, speed standard deviation, average longitudinal acceleration, maximum longitudinal acceleration, average lateral acceleration, and maximum lateral acceleration) extracted from each complete vehicle trajectory were directly used as input for clustering without transformation, as these aggregated statistical features already represent trajectory-level characteristics. Considering the heterogeneous and overlapping nature of driving behaviors in weaving areas, GMM was selected for its ability to provide probabilistic cluster assignments with uncertainty measures and accommodate clusters of varying shapes and sizes [19]. Unlike hard clustering methods such as K-means that assume spherical clusters of similar sizes, GMM’s flexible covariance structure is particularly appropriate for behavioral data where individuals may exhibit characteristics between different styles. The method also enables data-driven determination of optimal cluster numbers through information criteria. GMM is a probability-based clustering method that assumes data is composed of a mixture of multiple Gaussian distributions:


$$p(x)=\sum_{k=1}^{K}\pi_{k}N(x|\mu_{k},\Sigma_{k})$$ (3)

where $\pi_{k}$ represents the mixing weights, and $\mu_{k}$ and $\Sigma_{k}$ represent the mean and covariance matrix of the k-th Gaussian component, respectively. The optimal number of clusters was determined through Akaike Information Criterion (AIC) and Bayesian Information Criterion (BIC) information criteria, parameter estimation was performed using the Expectation-Maximization (EM) algorithm, and Principal Component Analysis (PCA) was combined for feature dimensionality reduction and visualization.

### Population distribution characteristic quantification method

A population distribution characteristic quantification indicator system was established, employing three complementary indicators to quantify the population distribution characteristics of driving styles. Shannon entropy, originating from information theory, is used to measure the uncertainty or balance of distribution; the Gini coefficient, originating from economics, reflects the degree of inequality in distribution; and the dominance index directly reflects the concentration degree of the dominant type:

Shannon entropy:


$$H=-\sum_{i=1}^{n}p_{i}\log p_{i}$$ (4)

Gini coefficient:


$$G=1-\sum_{i=1}^{n}p_{i}^{2}$$ (5)

Dominance index:


$$D=\max(p_{i})$$ (6)

where $p_{i}$ represents the proportion of the i-th driving style, and n represents the number of driving style types. Higher Shannon entropy values indicate more balanced distribution, larger Gini coefficients indicate more unequal distribution, and larger dominance index values indicate more concentrated dominant styles. The relationship patterns of operating parameters on population distribution were revealed through correlation analysis, analysis of variance, and regression modeling methods.

### Machine learning classification modeling

To establish robust driving style classification models and identify key influencing factors, five classic machine learning algorithms were employed: Logistic Regression for multi-class linear modeling, Random Forest for ensemble learning, XGBoost for gradient boosting, and Neural Network for deep learning classification. The mathematical formulations of these models are as follows:

Logistic Regression: Multi-class linear model


$$P(y=k|x)=\frac{e^{\beta_{k}^{T}x}}{\sum_{j=1}^{K}e^{\beta_{j}^{T}x}}$$ (7)

where $\beta_{k}$ represents the weight vector for the k-th class, K represents the number of categories, and x represents the feature vector.

Random Forest: Ensemble learning method


$$\hat{y}=\frac{\text{1}}{B}\sum_{b=1}^{B}T_{b}(x)$$ (8)

where B represents the number of decision trees, and $T_{b}(x)$ represents the prediction result of the b-th decision tree.

XGBoost: Gradient boosting decision tree


$$\hat{y}=\sum_{k=1}^{K}f_{k}(x),\;f_{k}\in F$$ (9)

where $f_{k}$ represents the k-th base learner (decision tree), and F represents the function space composed of all possible decision trees.

Neural Network: Multi-layer perceptron


$$y=f\left(W_{2}\cdot ReLU(W_{1}x+b_{1})+b_{2}\right)$$ (10)

where $W_{1}$ and $W_{2}$ represent the weight matrices from input layer to hidden layer and from hidden layer to output layer respectively, $b_{1}$ and $b_{2}$ represent the corresponding bias vectors, and ReLU is the activation function.

Prior to model training, all numerical features were standardized using z-score normalization to ensure comparable scales, with standardization parameters from the training set applied to the test set. For the neural network, L2 regularization (λ = 0.001), dropout (rate = 0.3), and early stopping (patience = 10 epochs monitoring validation loss) were employed to prevent overfitting. XGBoost used built-in regularization with max_depth = 6, min_child_weight = 1, and learning_rate = 0.1, while Random Forest was configured with 100 trees and max_depth = 15.

The dataset was divided into training and testing sets at an 8:2 ratio, and 5-fold cross-validation was employed to evaluate model generalization performance. Key factors affecting driving style classification were identified through feature importance analysis, quantifying the relative contributions of trajectory behavioral features, operating parameters, and path type features, providing a basis for establishing quantitative relationships between operating parameters and driving style distribution.

## Results

### Driving style identification and population distribution characteristics

#### Driving style identification results.

Based on drone trajectory data from 12,707 vehicle samples, GMM clustering analysis was performed on 6 driving style features. The optimal number of clusters was determined to be 7 through AIC and BIC information criteria, with an average highest probability of 0.857 for clustering results, indicating high classification certainty. Only 10.8% of samples showed classification uncertainty (probability < 0.6), demonstrating good clustering quality.

However, detailed examination of the 7 clusters revealed substantial behavioral overlap and similarity patterns. To determine the appropriate aggregation, three criteria were applied: (1) behavioral coherence – clusters exhibiting similar patterns across all features rather than just magnitude differences; (2) practical interpretability – alignment with established driving style classifications widely adopted in traffic behavior research [9]; and (3) statistical distinctiveness – ensuring aggregated groups maintain clear separation in feature space. Hierarchical analysis of the 7 cluster centroids in the 6-dimensional feature space confirmed that several clusters could be meaningfully grouped based on their proximity and similar behavioral profiles.

Based on these criteria, the 7 clusters were aggregated into 3 typical driving style types as shown in Table 2. Clusters 0, 1, and 5 were grouped as “moderate” due to their balanced performance across all metrics with moderate speed (58.1–71.3 km/h) and controlled acceleration levels. Clusters 2, 3, and 4 were categorized as “aggressive” characterized by either high speeds (>70 km/h), elevated longitudinal accelerations (>0.39 m/s²), or high lateral accelerations (>0.41 m/s²), reflecting more intense driving operations. Cluster 6 formed the “conservative” type, distinguished by its exceptionally low lateral acceleration (0.12 m/s²) compared to all other clusters, indicating highly cautious lateral maneuvering behavior.

**Table 2:** Summary of Driving Style Identification Results.

Cluster ID	Driving Style	Sample Size	Proportion (%)	Average Speed (km/h)	Speed Standard Deviation	Longitudinal Acceleration (m/s²)	Lateral Acceleration (m/s²)
0	moderate	2068	16.3	60.5	7.49	0.531	0.208
1	moderate	2798	22	71.3	1.4	0.254	0.299
2	Aggressive	1639	12.9	81	2.69	0.397	0.452
3	Aggressive	142	1.1	74.4	9.45	0.998	0.497
4	Aggressive	1204	9.5	57.5	4.9	0.417	0.417
5	moderate	2623	20.6	58.1	3.67	0.341	0.262
6	Conservative	2233	17.6	68.5	3.54	0.291	0.12
Total/Average	–	12707	100	66.3	3.83	0.366	0.278

From the clustering results, it can be observed that aggressive driving style accounts for 23.5%, with main characteristics being high speed (average 71.2 km/h) and high acceleration operation intensity (longitudinal 0.604 m/s², lateral 0.455 m/s²). moderate driving style dominates (58.9%), with moderate levels across all indicators, demonstrating relatively balanced driving characteristics. Conservative driving style accounts for 17.6%, with the prominent feature being extremely low lateral acceleration (0.120 m/s²), reflecting high caution in lateral operations.

**Fig 1:**
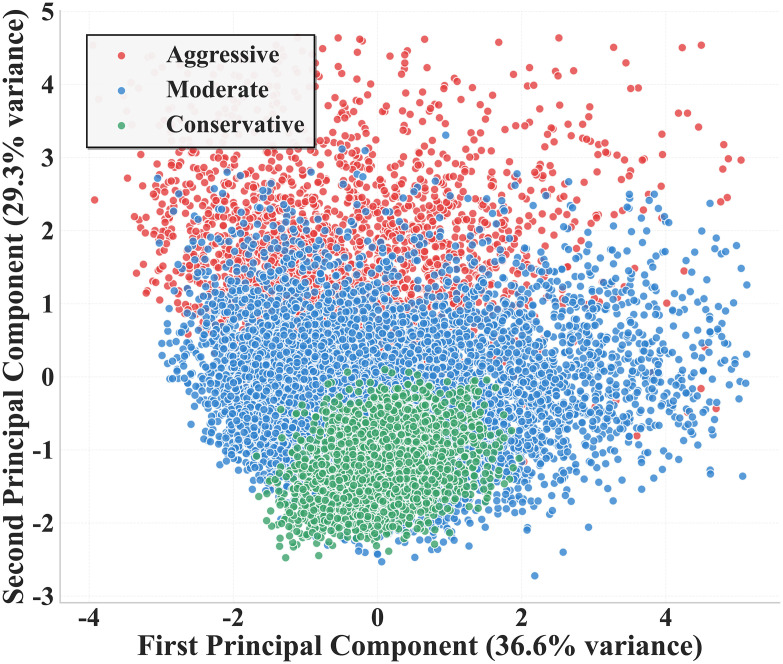
PCA Visualization of Driving Style Clusters.

Fig 1 shows the two-dimensional spatial distribution of driving style clustering results after outlier removal in PCA-reduced dimensions. Through PCA, the first principal component explained 36.6% of the variance, and the second principal component explained 29.3% of the variance. The two principal components cumulatively explained approximately 66% of the total variance, indicating good dimensionality reduction performance. Three distinct spatial distribution patterns of driving styles can be clearly observed in the feature space: aggressive driving style (red) is mainly distributed in the upper half of the figure, showing higher values of the second principal component; conservative driving style (green) is concentrated in the lower-left quadrant, exhibiting lower values for both the first and second principal components; moderate driving style (blue) is widely distributed in the central area and right side, occupying the main portion of the feature space. Although there is some degree of overlap among the three driving styles in boundary regions, the overall separation effect is good, validating the effectiveness of GMM clustering methods for driving style identification.

**Fig 2:**
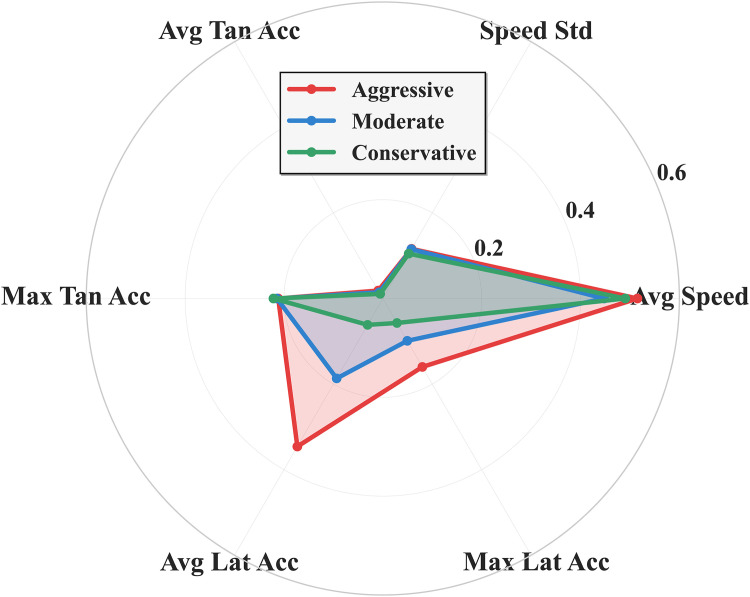
Driving Style Characteristics Comparison.

Fig 2 compares the standardized performance of three driving styles across 6 key features using radar charts in the same coordinate system. Aggressive driving style (red) shows outstanding performance in Avg Tan Acc and Max Tan Acc dimensions, significantly higher than other types, but relatively lower in Avg Speed, forming an irregular contour. Conservative driving style (green) shows stable and low values across all feature dimensions, particularly displaying the most conservative characteristics in Avg Lat Acc and Max Lat Acc, with the radar chart presenting a compact shape. moderate driving style (blue) shows relatively balanced indicators, with contours between aggressive and conservative types, slightly higher than other types in Avg Speed and Speed Std, reflecting moderate and stable driving characteristics. The differentiated characteristics of the three driving style radar charts validate the effectiveness of the clustering analysis.

#### Population distribution characteristic analysis.

The driving style population distributions across weaving zones show significant differences, as shown in Table 3. Zone 3 has the highest proportion of aggressive type (65.7%), while Zones 4 and 5 have the highest proportions of conservative type (66.9% and 67.5%, respectively). These distribution differences are closely related to the operating parameters of each weaving zone, with short weaving zones and high speed limit areas tending to attract more aggressive driving styles, while long weaving zones and low speed limit areas form clusters of conservative driving styles.

**Table 3:** Distribution Characteristics of Driving Style Groups Across Weaving Zones.

Zone	Aggressive (%)	Moderate (%)	Conservative (%)	Shannon Entropy	Gini Coefficient	Dominance Index	Dominant Pattern
1	21.4	78.5	0.1	0.524	0.338	0.785	Moderate
2	23.4	75.8	0.8	0.589	0.371	0.758	Moderate
3	65.7	34.3	0	0.643	0.451	0.657	Aggressive
4	0.8	32.4	66.8	0.671	0.448	0.669	Conservative
5	1.9	30.6	67.5	0.703	0.45	0.675	Conservative
6	13.3	86.1	0.6	0.431	0.242	0.86	Moderate
7	12.1	87.8	0.1	0.376	0.215	0.878	Moderate

Quantitative indicators of population distribution show that Shannon entropy values range from 0.376 to 0.703, reflecting the degree of heterogeneity in population distributions across different weaving zones. Zone 5 has the highest Shannon entropy (0.703), indicating its driving style distribution is the most balanced; Zone 7 has the lowest Shannon entropy (0.376), indicating that a single driving style holds absolute dominance. The Gini coefficient shows positive correlation with Shannon entropy, ranging from 0.215 to 0.451, further confirming the differentiated characteristics of population distribution.

**Fig 3:**
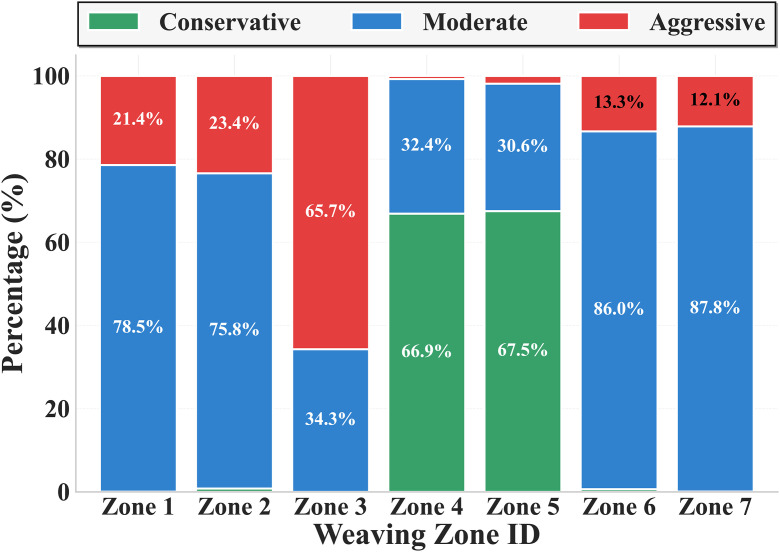
Driving Style Distribution Across Weaving Zones.

Fig 3 intuitively displays the driving style distribution differences across 7 weaving zones in the form of stacked bar charts. Significant distribution differences among weaving zones can be clearly observed from the figure: Zones 1, 2, 6, and 7 are dominated by moderate type (blue portion dominates), Zone 3 shows aggressive type dominance characteristics (red portion exceeds 60%), and Zones 4 and 5 are dominated by conservative type (green portion dominates). This differentiated distribution pattern provides intuitive data support for subsequent analysis of operating parameter relationship patterns.

**Fig 4:**
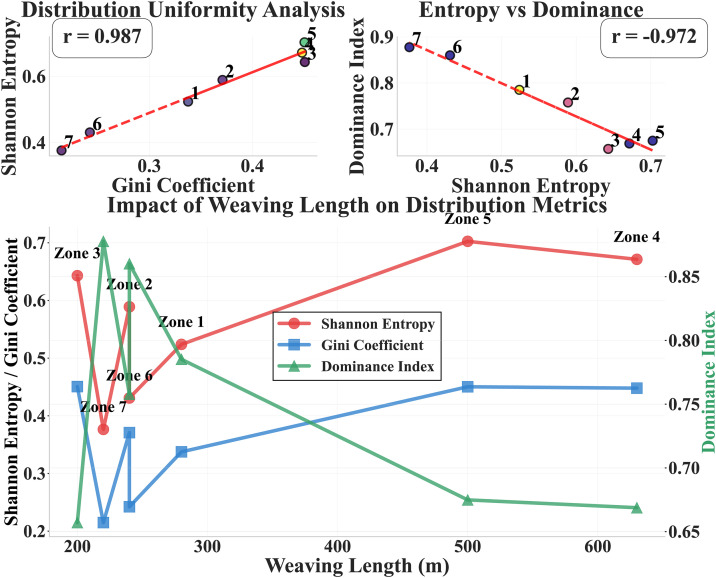
Distribution Metrics Comprehensive Analysis.

Fig 4 shows the correlations among population distribution quantification indicators and the relationships with operating parameters through scatter plot matrices and line charts. The upper two scatter plots show: Shannon entropy has a strong positive correlation with Gini coefficient (r = 0.987), indicating high consistency between these two indicators in measuring distribution balance; Shannon entropy has a strong negative correlation with dominance index (r = −0.972), which meets theoretical expectations that the more balanced the distribution, the lower the dominance of the leading style. The lower line chart shows the relationship of weaving length on distribution indicators: as weaving length increases from 200m to 600m, Shannon entropy shows an overall upward trend (from approximately 0.38 in Zone 7 to approximately 0.67 in Zone 4), Gini coefficient shows a similar change pattern, while dominance index shows a downward trend (from approximately 0.88 in Zone 7 to approximately 0.67 in Zone 4). It is noteworthy that there are large indicator fluctuations in short weaving zones of 200-300m (Zones 1, 2, 6, 7), while indicators tend to stabilize in longer weaving zones (Zones 4, 5), indicating that weaving length has significant systematic influence on driving style population distribution.

#### Impact of path type and vehicle type on driving style distribution.

The driving style distribution characteristics for different path types and vehicle types are shown in Table 4. Through-mainline vehicles account for 54.5% of the total sample and are dominated by moderate type; merging vehicles account for 21.5% with relatively high proportion of aggressive type; diverging vehicles account for 17.4%, with driving style distribution significantly influenced by weaving zone operating parameters. In terms of vehicle types, passenger cars have the highest proportion of aggressive type, while large vehicles tend more toward conservative driving styles, which aligns with vehicle power performance and handling characteristics.

**Table 4:** Driving Style Distribution Characteristics by Path Type and Vehicle Type.

Classification Dimension	Category	Sample Size	Proportion (%)	Aggressive (%)	Moderate (%)	Conservative (%)	Main Characteristics
Path Type	Through-mainline	6924	54.5	18.2	71.4	10.4	Moderate-dominated with stable distribution
	Merging	2727	21.5	32.8	58.1	9.1	High proportion of aggressive type
	Diverging	2213	17.4	26.1	45.7	28.2	Significantly influenced by operating parameters
	Through-ramp	843	6.6	15.4	68.9	15.7	Similar to through-mainline
Vehicle Type	Passenger car	11101	87.4	25.1	58.8	16.1	Highest proportion of aggressive type
	Medium vehicle	1178	9.3	16.2	63.2	20.6	Relatively conservative
	Heavy vehicle	346	2.7	8.7	55.5	35.8	High proportion of conservative type
	Others	82	0.6	12.2	67.1	20.7	Small sample size

### Relationships between operating parameters and driving style distribution

**Fig 5:**
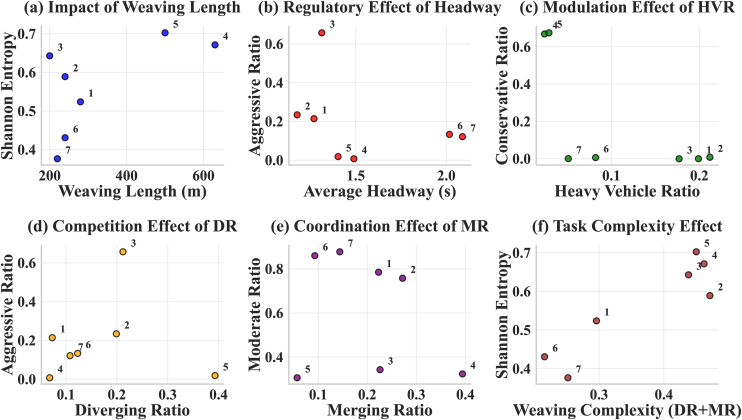
Parameter-Distribution Relationship Analysis.

To reveal the influence patterns of weaving zone operating parameters on driving style population distribution, the study constructed a multi-level analysis framework, systematically analyzing the relationships from three dimensions: geometric operating parameters, traffic operating parameters, and weaving task parameters. Fig 5 shows the quantitative relationships between six key parameters and driving style distribution characteristics, with each subplot presenting the distribution patterns of 7 weaving zones under different parameter combinations in scatter plot form. Fig 5a shows the relationship of weaving length on Shannon entropy, Fig 5b reflects the relationship between headway time and aggressive type proportion, Fig 5c displays the association with HVR on conservative type proportion, Fig 5d and e respectively demonstrate the differentiation effects of DR and MR on corresponding driving styles, and Fig 5f presents the systematic relationship between weaving complexity and Shannon entropy. Each weaving zone is identified by Zone number in the figures. Additionally, the study defines weaving complexity as the sum of DR and MR (Weaving Complexity = DR + MR), which comprehensively reflects the overall task intensity of vehicles completing diverging and merging operations within the weaving zone.

#### Fundamental role of geometric operating parameters.

Fig 5a clearly demonstrates the relationship pattern of weaving length on Shannon entropy, with the 7 weaving zones showing distinct stratified distribution characteristics in the coordinate system. Short weaving zones (Zones 1, 2, 3, 6, 7) are mainly distributed in the left low-entropy region, with Shannon entropy values ranging from 0.376 to 0.643, while long weaving zones (Zones 4, 5) are located in the right high-entropy region, with Shannon entropy reaching 0.671 and 0.703, respectively. Statistical analysis shows that weaving length has a moderate positive correlation with Shannon entropy (r = 0.629, p = 0.131). Although the p-value does not reach the traditional significance level, the correlation coefficient is large and the actual effect is evident.

Table 5 details the analysis results grouped by length. Based on the current dataset, 300m appears to represent a potential transition point: short weaving zones (<300m) contain 5 weaving zones with an average Shannon entropy of only 0.513, indicating that driving style distribution tends toward homogenization; long weaving zones (≥300m) contain Zones 4 and 5 with an average Shannon entropy of 0.687, significantly higher than short weaving zones. The significance test results (t = −2.112, p = 0.088) are at a marginally significant level, but the practical difference is notable. However, it should be noted that this observation is based on a limited sample of seven weaving areas, with only two zones in the longer length category, and requires further validation.

**Table 5:** Relationship between Weaving Length and Driving Style Distribution.

Length Group	Zone Numbers	Number of Zones	Average Shannon Entropy	Main Characteristics
Short (<300m)	1,2,3,6,7	5	0.513	Homogenized style distribution
Medium (300-500m)	5	1	0.703	Most balanced distribution
Long (>500m)	4	1	0.671	Relatively balanced distribution

In short weaving zones, limited space appears to lead to more homogenized driving behaviors, while longer zones allow for greater behavioral diversity.

#### Moderating effects of traffic operating parameters.

Fig 5b shows the relationship between headway time and aggressive driving style proportion. More importantly, there is a strong negative correlation between headway time and Shannon entropy (r = −0.760, p = 0.047), which is the strongest and statistically significant correlation among all parameters in this study. From the figure, it can be observed that as headway time increases, weaving zones exhibit distinct stratified patterns, with low headway time areas (Zones 1, 2) having relatively high aggressive type proportions, while high headway time areas (Zones 6, 7) show significantly reduced aggressive type proportions.

Table 6 presents detailed analysis results grouped by traffic density, revealing the complex relationships between density and behavioral patterns. Under high-density traffic conditions (headway time < 1.3s, including Zones 1, 2), the average Shannon entropy is 0.556, with aggressive type proportion averaging 22.4% and moderate type proportion at 77.1%. Frequent vehicle interactions and limited maneuvering space suppress extreme driving behaviors, but the dynamic nature of traffic flow still provides some expression space for different styles. Under medium-density conditions (headway time 1.3−1.6s, including Zones 3, 4, 5), there is the highest distribution balance, with average Shannon entropy reaching 0.672. This density range provides moderate expression space for various driving styles, neither excessively constraining aggressive behavior nor lacking necessary traffic flow constraints. Under low-density conditions (headway time > 1.6s, including Zones 6, 7), an anomalous phenomenon occurs, with average Shannon entropy dropping to 0.404, aggressive type proportion only 12.7%, and moderate type proportion as high as 86.9%, indicating that lack of traffic flow constraints actually leads to homogenization of driving behavior.

**Table 6:** Association between Traffic Density and Driving Style Distribution.

Density Group	Headway Range	Zone Numbers	Average Shannon Entropy	Aggressive Proportion (%)	Moderate Proportion (%)
High density	<1.3s	1,2	0.556	22.4	77.1
Medium density	1.3−1.6s	3,4,5	0.672	22.8	31.4
Low density	>1.6s	6,7	0.404	12.7	86.9

Fig 5c shows the relationship between HVR and conservative driving style proportion, exhibiting strong inverse correlation (r = −0.694, p = 0.084). Zones 4 and 5 have the lowest HVR (2.4% and 2.9%, respectively) but show the highest conservative type proportions (66.9% and 67.5%), located in the upper-left area of the figure. Zones 1, 2, and 3 with higher HVR (average 19.6%) have conservative type proportions close to zero, distributed in the lower-right area. Table 7 details the differentiated impacts of HVR on different driving styles, showing the indirect relationship pattern of HVR through changing traffic flow microstructure. In high HVR environments, the presence of heavy vehicles provides more following and lane-changing opportunities for passenger car drivers. The relatively low acceleration performance and larger body size of heavy vehicles create a relatively stable traffic flow environment, stimulating aggressive driving behavior among passenger car drivers. In low HVR environments, direct competition among passenger cars intensifies, with drivers facing more competition from similar-performance vehicles, which conversely prompts them to adopt more cautious conservative strategies.

**Table 7:** Association between HVR and Driving Style Distribution.

HVR Group	Zone Numbers	Average HVR (%)	Conservative Proportion (%)	Aggressive Proportion (%)	Moderate Proportion (%)
High (>15%)	1,2,3	19.6	0.3	38.2	61.5
Medium (8–15%)	6	8.2	0.6	13.3	86
Low (<8%)	4,5,7	3.5	44.8	6.8	48.4

#### Differentiation effects of weaving task parameters.

Fig 5f presents the most important finding of this study: the strong positive correlation between weaving complexity (DR + MR) and Shannon entropy (r = 0.909, p = 0.005), which is a statistically highly significant result. The correlation coefficient approaching 0.9 indicates a near-linear strong correlation between the two variables. From the figure, it can be observed that the 7 weaving zones are distributed almost perfectly along a diagonal line from lower-left to upper-right, with Zone 5 located in the upper-right corner (complexity 0.45, entropy 0.703), Zone 7 in the lower-left corner (complexity 0.252, entropy 0.376), and the remaining weaving zones distributed sequentially along this diagonal, forming a clear linear relationship pattern.

Table 8 shows the systematic relationship between weaving complexity and driving style distribution, showing the multi-level associations of complexity on driving style distribution. Zone 7 with the lowest complexity (0.252) exhibits the strongest single-style dominance characteristic, with moderate type proportion as high as 87.8% and Shannon entropy only 0.376, reflecting high consistency of driving behavior under simple weaving tasks. Zone 5 with the highest complexity (0.450), although dominated by conservative type (67.5%), has Shannon entropy reaching the highest value of 0.703, indicating that complex weaving tasks create differentiation space for different driving style expressions. Medium-complexity weaving zones present different dominance patterns: Zone 3 (complexity 0.438) forms aggressive type dominance (65.7%), while Zone 4 (complexity 0.462) forms conservative type dominance (66.9%). These differences mainly stem from different combination patterns of DR and MR.

**Table 8:** Systematic Relationship between Weaving Complexity and Driving Style Distribution.

Zone	DR (%)	MR(%)	Complexity	Shannon Entropy	Dominant Style	Dominant Proportion (%)
7	10.8	14.4	0.252	0.376	Moderate	87.8
6	12.3	9.3	0.216	0.431	Moderate	86
1	7.3	22.3	0.296	0.524	Moderate	78.5
2	19.9	27.2	0.471	0.589	Moderate	75.8
3	21.2	22.6	0.438	0.643	Aggressive	65.7
4	6.8	39.4	0.462	0.671	Conservative	66.9
5	39.3	5.7	0.45	0.703	Conservative	67.5

Fig 5d and e respectively show the independent effects of DR and MR. The correlation between DR and aggressive type proportion is weak (r = 0.040, p = 0.933), but in Fig 5d, Zone 3 can be observed to form obvious aggressive type clustering, with 21.2% DR corresponding to 65.7% aggressive type proportion, appearing as an isolated point in the upper-right corner of the figure. This clustering phenomenon indicates that although the overall correlation between DR and aggressive type proportion is not strong, significant local effects can still occur under specific conditions. Fig 5e shows that MR has negative correlation with moderate type proportion (r = −0.250, p = 0.589), with Zone 5’s extremely low MR (5.7%) corresponding to lower moderate type proportion (30.6%), while Zone 4’s high MR (39.4%) corresponds to lower moderate type proportion (32.4%).

The analysis shows different patterns for diverging and merging tasks, with DR associated with more aggressive behaviors and MR showing different correlational patterns.

### Quantitative modeling of the relationship between operating parameters and distribution

#### Modeling data and feature construction.

Based on the clustering analysis results from the previous section, this study used 12,707 complete trajectory data as modeling samples, including 7,489 moderate driving style samples (59.0%), 2,985 aggressive samples (23.5%), and 2,233 conservative samples (17.6%). To comprehensively characterize driving behavioral features and weaving zone environmental characteristics, a multidimensional feature system was constructed containing trajectory behavioral features, weaving zone operating parameters, and path type features.

The detailed information of the constructed feature system is shown in Table 9. Trajectory behavioral features directly reflect vehicle motion characteristics, weaving zone operating parameters embody objective environmental conditions of weaving zones, and path type features are processed through one-hot encoding to reflect different weaving behavioral patterns.

**Table 9:** Feature System for Driving Style Classification.

Feature Category	Feature Name	Description	Quantity
Trajectory Behavioral Features	avg_speed	Average speed	6
	speed_std	Speed standard deviation	
	avg_abs_tan_acc	Average longitudinal acceleration	
	max_tan_acc	Maximum longitudinal acceleration	
	avg_abs_lat_acc	Average lateral acceleration	
	max_lat_acc	Maximum lateral acceleration	
Weaving Zone Operating Parameters	weaving_length	Weaving length	7
	speed_limit	Speed limit	
	avg_headway	Average headway	
	HVR	/	
	DR	/	
	MR	/	
	weaving_complexity	Weaving complexity	
Path Type Features	through_main	Through-mainline (54.5%)	4
	merging	Merging path (21.5%)	
	diverging	Diverging path (17.4%)	
	through_ramp	Through-ramp (6.6%)	

#### Machine learning model performance comparison.

Four classic machine learning algorithms were employed to construct driving style classification models: logistic regression, random forest, XGBoost, and neural network. The dataset was divided into training set (10,165 samples) and test set (2,542 samples) at an 8:2 ratio, with 5-fold cross-validation used to evaluate model generalization performance. The results in Table 10 show that the neural network model performed best, achieving 95.4% test accuracy and 95.3% ± 0.3% cross-validation accuracy, demonstrating excellent classification performance and stability. XGBoost followed closely with 94.7% test accuracy. In contrast, the traditional logistic regression model achieved only 85.1% accuracy, indicating that driving style classification problems have strong nonlinear characteristics requiring more complex model structures to capture interactions among features.

**Table 10:** Performance Comparison of Machine Learning Models.

Model	Test Accuracy	Cross-validation Mean	Cross-validation Standard Deviation
Logistic Regression	0.851	0.848	0.003
Random Forest	0.929	0.929	0.005
XGBoost	0.947	0.952	0.004
Neural Network	0.954	0.953	0.003

#### Feature importance analysis.

Feature importance analysis was conducted using the XGBoost model rather than the best-performing neural network (95.4% vs 94.7% accuracy) due to interpretability considerations. XGBoost provides direct and reliable feature importance scores through its built-in gain calculation mechanism, whereas neural networks require complex post-hoc interpretation methods that may be less stable with the current dataset. Given the minimal performance difference and the research objective of understanding how operational parameters influence driving style classification, the interpretable tree-based approach was prioritized.

**Fig 6:**
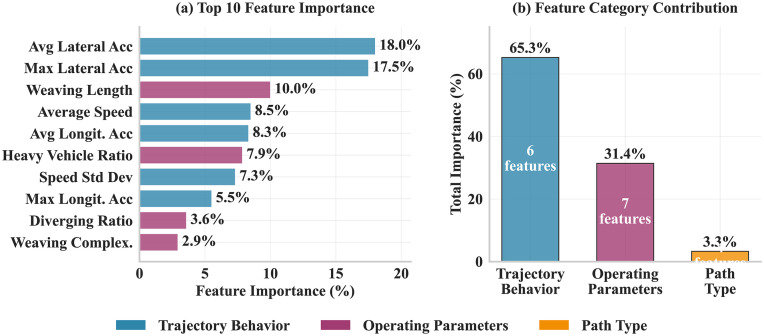
Feature Importance Analysis.

Feature importance analysis based on the XGBoost model revealed key factors affecting driving style classification and their relative contributions. Fig 6 shows the contribution ranking of the top 10 important features and the overall importance distribution of different feature categories.

Analysis results show that trajectory behavioral features dominate with total importance reaching 65.3%. Among these, average lateral acceleration (18.0%) and maximum lateral acceleration (17.5%) rank first and second, contributing a combined 35.5%, indicating that lateral motion characteristics are the most critical indicators for distinguishing driving styles. This finding aligns with driving behavioral characteristics in weaving zones: aggressive drivers tend to change lanes frequently and make sharp turns, generating greater lateral acceleration; while conservative drivers change lanes cautiously with relatively smooth lateral movements.

Weaving zone operating parameters have 31.4% importance, reflecting the significant impact of infrastructure conditions on driving style formation. Weaving length (10.0%) is the most important operating parameter, demonstrating the strong associations between geometric conditions and driving behavior. Traffic flow parameters such as HVR (7.9%) and DR (3.6%) also have important impacts, reflecting the moderating effect of traffic environment complexity on driving style selection. Path type features have relatively low importance (3.3%), mainly because path types are already covered to some extent by other features, but still contribute positively to model performance.

## Discussion

This study advances understanding of weaving area operations by shifting focus from individual driving style identification to population distribution analysis. The strong positive correlation between weaving complexity and Shannon entropy (r = 0.909, p = 0.005) reveals that task complexity systematically influences behavioral diversity in transportation systems. Higher weaving complexity, characterized by increased diverging and merging demands, creates multiple competing objectives for drivers, activating different behavioral strategies and resulting in more heterogeneous population distributions. This finding suggests that weaving area design parameters can be strategically used to influence the composition of driver populations, though such applications require careful safety considerations [20].

The multi-level relationship patterns identified in this research form a hierarchical system where geometric, traffic flow, and task parameters operate at different scales. Geometric parameters, particularly the identified 300m weaving length threshold, establish fundamental spatial constraints that determine whether drivers can express individual style preferences or are forced into homogenized behaviors. Traffic flow parameters generate dynamic regulation effects, with the counterintuitive finding that moderate density conditions (headway 1.3−1.6s) promote higher behavioral diversity than either very high or very low density conditions. This non-linear relationship challenges conventional traffic management assumptions and suggests optimal density ranges for maintaining healthy population distributions.

The observed transition pattern around 300m weaving length aligns with established highway design standards. AASHTO design guidelines have historically recommended minimum weaving section lengths of approximately 300m (1000 ft) between successive ramp terminals, reflecting the spatial requirements for drivers to perceive gaps, make decisions, and complete weaving maneuvers safely. Shorter sections (<300m) may constrain behavioral choices and force more uniform strategies, while longer sections (≥300m) provide space for individual style expression. However, given the limited observation sites, this interpretation remains preliminary and requires further validation.

From a methodological perspective, the dominant role of lateral acceleration features (35.5% combined importance) validates the significance of lateral operations in weaving areas and provides a practical foundation for real-time monitoring systems. The 95.4% identification accuracy achieved by machine learning models, combined with the finding that operating parameters account for 31.4% of feature importance, demonstrates that driving style is context-dependent rather than purely individual. This contextual dependency has important implications for both research methodology and practical applications, suggesting that environmental parameters should be standard components of behavioral models.

The research findings enable a conceptual shift from reactive to proactive safety management approaches. Population distribution monitoring could identify emerging risk patterns before they manifest as accidents, particularly when behavioral diversity decreases to critical levels. However, practical implementation faces significant challenges including infrastructure costs, technological validation under diverse conditions, and the need for comprehensive cost-benefit analyses. The integration with emerging connected vehicle technologies presents opportunities for more sophisticated monitoring systems, though the transition period with mixed human-autonomous traffic streams will require new modeling approaches and management strategies [21].

The findings have important practical implications for intelligent transportation system development. The identified 300m weaving length threshold provides actionable guidance for geometric design, while the strong correlation between weaving complexity and Shannon entropy (r = 0.909, p = 0.005) suggests that real-time monitoring of behavioral diversity could serve as an early warning system for safety risks. The high classification accuracy (95.4%) demonstrates feasibility for implementing driving style identification through connected vehicle systems. Beyond weaving areas, this population distribution framework has broader significance as the methodology could be adapted to other conflict zones and the finding that operational parameters account for 31.4% of feature importance reveals that collective behavior patterns can be systematically shaped by infrastructure design. This is particularly relevant for mixed traffic environments where autonomous vehicles must coexist with human drivers, requiring understanding of population behavioral patterns for compatible algorithm design and representing a shift from reactive incident management to proactive risk prevention.

Several limitations should be acknowledged in this study. First, the analysis is based on data from only seven weaving areas, which may limit the generalizability of findings to other geometric configurations and traffic conditions. Particularly, the identification of 300m as a potential transition point is based on limited sampling and should be considered preliminary, requiring validation through additional field studies or simulation approaches. Second, the data collection was conducted under controlled environmental conditions (clear weather, daytime, good visibility) to ensure data quality and minimize confounding effects. However, driving behavioral patterns may vary under different weather, lighting, or time-of-day scenarios, and the interaction effects between environmental factors and operational parameters warrant investigation in future studies. Third, while the study establishes correlations between operational parameters and driving style distributions, causal relationships cannot be definitively established from observational data alone.

Future research should expand the analysis to include more diverse weaving configurations and traffic conditions to enhance the generalizability of findings. The 300m threshold observation could be further validated through simulation studies or expanded field data collection. Longitudinal studies examining how population distributions change over time and under different conditions would provide valuable insights into the stability of these patterns. Additionally, experimental studies using traffic simulation or controlled field experiments could help establish causal relationships between operational parameters and behavioral outcomes. Research into the practical implementation of population distribution monitoring systems, including cost-benefit analyses and technological feasibility assessments, would also contribute to the field.

## Conclusions

This study developed a quantitative analysis framework for examining the relationships between weaving area operational parameters and driving style population distribution, based on drone trajectory data from seven weaving areas. Through Gaussian Mixture Model clustering analysis, three driving style types were identified: aggressive (23.5%), moderate (58.9%), and conservative (17.6%). Population distributions across weaving areas showed significant variations, with Shannon entropy values ranging from 0.376 to 0.703.

The analysis revealed systematic relationships between operational parameters and population distribution patterns. The strong positive correlation between weaving complexity and Shannon entropy (r = 0.909, p = 0.005) suggests that task complexity is significantly associated with population distribution equilibrium. The identification of a 300m weaving length threshold and the regulatory effect of headway time confirmed the influence of geometric conditions and traffic flow characteristics on driving style distribution. The machine learning classification model achieved 95.4% identification accuracy, with lateral acceleration features showing the highest importance (35.5%) and operational parameters contributing 31.4% of feature importance.

These findings provide new perspectives for weaving area operation optimization and suggest potential applications for advanced traffic management systems. The driving style population distribution analysis offers insights that complement traditional individual-focused analyses, though practical implementation would require careful consideration of technological, economic, and operational factors. The research contributes to the understanding of how infrastructure and traffic conditions influence driver behavior patterns, with implications for both traffic engineering practice and intelligent transportation system development. Preliminary applications include geometric design guidance based on the identified 300m length threshold, real-time behavioral diversity monitoring for proactive safety management, and personalized warning systems enabled by the high classification accuracy achieved in this study.

## Supporting information

S1 File Combined_driving_style_features. (CSV)
